# Identification of ER-000444793, a Cyclophilin D-independent inhibitor of mitochondrial permeability transition, using a high-throughput screen in cryopreserved mitochondria

**DOI:** 10.1038/srep37798

**Published:** 2016-11-25

**Authors:** Thomas Briston, Sian Lewis, Mumta Koglin, Kavita Mistry, Yongchun Shen, Naomi Hartopp, Ryosuke Katsumata, Hironori Fukumoto, Michael R. Duchen, Gyorgy Szabadkai, James M. Staddon, Malcolm Roberts, Ben Powney

**Affiliations:** 1 UCL Collaboration Research Group, NGM-PCU, Eisai Ltd., Hatfield, UK; 2 Next Generation Systems CFU, Eisai Inc., Andover, MA, USA; 3 Next Generation Systems CFU, Eisai Co., Ltd, Tsukuba, Japan; 4 NGM-PCU, Eisai Co. Ltd., Tsukuba Research Laboratories, Tsukuba, Japan; 5 Department of Cell and Developmental Biology, University College London, London, UK; 6 Department of Biomedical Sciences, University of Padua, Padua, Italy

## Abstract

Growing evidence suggests persistent mitochondrial permeability transition pore (mPTP) opening is a key pathophysiological event in cell death underlying a variety of diseases. While it has long been clear the mPTP is a druggable target, current agents are limited by off-target effects and low therapeutic efficacy. Therefore identification and development of novel inhibitors is necessary. To rapidly screen large compound libraries for novel mPTP modulators, a method was exploited to cryopreserve large batches of functionally active mitochondria from cells and tissues. The cryopreserved mitochondria maintained respiratory coupling and ATP synthesis, Ca^2+^ uptake and transmembrane potential. A high-throughput screen (HTS), using an assay of Ca^2+^-induced mitochondrial swelling in the cryopreserved mitochondria identified ER-000444793, a potent inhibitor of mPTP opening. Further evaluation using assays of Ca^2+^-induced membrane depolarisation and Ca^2+^ retention capacity also indicated that ER-000444793 acted as an inhibitor of the mPTP. ER-000444793 neither affected cyclophilin D (CypD) enzymatic activity, nor displaced of CsA from CypD protein, suggesting a mechanism independent of CypD inhibition. Here we identified a novel, CypD-independent inhibitor of the mPTP. The screening approach and compound described provides a workflow and additional tool to aid the search for novel mPTP modulators and to help understand its molecular nature.

Mitochondrial Ca^2+^ accumulation is critically important for cellular homeostasis. The spatiotemporal regulation of Ca^2+^ by mitochondria drives diverse cellular functions ranging from control of oxidative metabolism to induction of cell death^1^,^2^,^3^,^4^. Failure in cellular Ca^2+^ homeostasis and consequent mitochondrial Ca^2+^ overload is the principal trigger for mitochondrial permeability transition (mPT)^5^,^6^,^7^. mPT defines a sudden increase in mitochondrial inner membrane permeability to low molecular weight solutes of less than 1500 Daltons^7^. Stress-induced opening of a voltage- and Ca^2+^- sensitive, high conductance inner membrane channel, the ‘mitochondrial permeability transition pore’ (mPTP) is associated with matrix swelling, dissipation of mitochondrial membrane potential, uncoupling of oxidative phosphorylation and cellular metabolic insufficiency^6^,^8^,^9^,^10^. Growing evidence suggests that persistent mPTP opening is a key pathophysiological event in cellular death underlying a wide variety of human diseases and disorders, notably ischaemia-reperfusion injury of the heart and brain^11^,^12^,^13^,^14^, neurodegeneration^15^,^16^ and muscular dystrophies^17^,^18^. The development of mPTP inhibitors is therefore warranted, as new agents could have a wide range of therapeutic applications in the clinic and also have utility in understanding the biomolecular nature of the pore itself.

Cyclophilin D (CypD), although not an integral pore component, is a primary positive regulator of mPTP opening^19^,^20^. Pharmacological inhibition or genetic ablation of CypD enzymatic activity desensitises the pore, thereby reducing the probability of pore opening and increasing mitochondrial Ca^2+^ tolerance^21^,^22^,^23^,^24^,^25^. Therapeutic targeting of CypD is therefore somewhat limited, as its effects on pore opening are indirect and mitochondria remain ultimately capable of permeability transition^21^,^25^. To date cyclosporin A (CsA) is the best characterised inhibitor of the mPTP, exerting its effect by inhibiting CypD^22^,^24^,^26^. However, CsA demonstrates lack of selectivity for inhibiting CypD over other cyclophilins (consisting of 16 family members^27^) and shows a strong immunosuppressive effect in humans, restricting its therapeutic development potential for treating mitochondrial dysfunction^28^,^29^,^30^. The challenges of selectively targeting CypD are clear and therefore identification of CypD-independent mPTP inhibitors is desirable^31^. Despite identification of small molecule inhibitors of mPT presenting an obvious therapeutic opportunity, the availability and development of such agents remains limited. A number of groups have identified novel molecules modulating mitochondrial propensity for permeability transition^30^,^32^,^33^,^34^,^35^,^36^,^37^. However, as yet, reports of positive clinical development are yet to emerge.

In order to screen for novel mPTP inhibitors in a rapid and efficient manner, we exploited and finessed a method to isolate mitochondria and preserve function after freeze-thaw using the cryopreservative agent trehalose^38^. A high-throughput screen (HTS) to identify new inhibitors of mPT was then performed using the trehalose-stabilised mitochondria. This HTS yielded a number of compounds of interest. To investigate mechanism of action of the compounds, a panel of assays (mitochondrial swelling, Ca^2+^-induced mitochondrial membrane depolarisation and Ca^2+^ retention capacity) was deployed. This enabled identification of compounds specifically modulating mPT and eliminated compounds dissipating membrane potential and therefore inhibiting mitochondrial Ca^2+^ uptake. Further studies were performed to understand whether the compounds had CypD-dependence. Compounds were also investigated for general effects on mitochondrial and cellular health.

As a result of these studies, we now describe the identification, validation and characterisation of N-(2-benzylphenyl)-2-oxo-1H-quinoline-4-carboxamide (ER-000444793), a small molecule, non-toxic mPTP inhibitor with a mechanism of action independent of CypD inhibition.

## Results

### Validating the functionality of cryopreserved mitochondria by high resolution respirometry and ATP synthesis

Trehalose-preserved mitochondria have previously been demonstrated to retain function, albeit with some respiratory compromise after storage at −80 °C^38^. To assess the utility of this approach, we isolated mitochondria from rat liver using a slightly modified method from that published by Yamaguchi *et al*.^38^. To use cryopreserved mitochondria for high-throughput screening, isolation was also scaled-up, such that single batches of 30–35 ml of 50 mg ml^−1^ protein were prepared from 12 rat livers at a single time. A total of 20 batches were prepared and stored in trehalose in 0.5–1 ml aliquots at −80 °C. Following quality control (QC) analysis using the Ca^2+^-induced mitochondrial swelling assay, three batches were discarded as they failed to produce Z′ > 0.35 using CsA (5 μM) and DMSO as positive and negative controls respectively. Z′ defines a screening window coefficient and is illustrative of assay quality. It is representative of both the assay window (i.e. separation between positive and negative controls) and the variation of assay measurements (for equation – see Methods). For our screening campaign and subsequent hit assessment, assay consistency was extremely important and so mitochondrial quality and assay performance (shown in Table 1) was assessed over time. Here, the Z′ measure of assay quality was calculated over a 7 month period of analogue follow-up screening in the Ca^2+^ retention assay. Z′ values were maintained above 0.37 over the 7 month period, suggesting mitochondrial activity and preparation quality is suitably retained after freeze-thaw using the method described.

**Figure 1:**
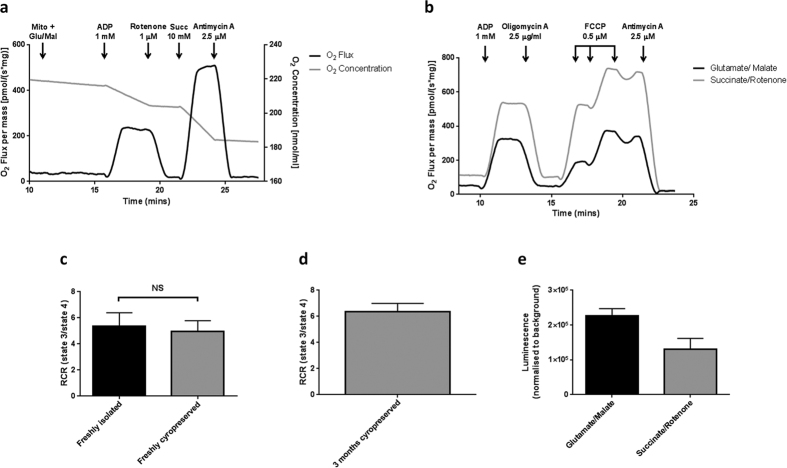
Bioenergetic validation of cryopreserved mitochondrial preparations. (**a**) Representative oxygen flux recording using closed-chamber, high-resolution respirometry. Rat liver mitochondria (0.25 mg protein ml^−1^) were maintained under constant stirring. Oxygen consumption (black trace) and oxygen concentration (grey trace) were measured using the Oxygraph 2 K (Oroboros Instruments, Innsbruck, Austria). Sequential additions of reagents were added to assess respiratory states. (**b**) Mitochondria energised using either glutamate/malate (10 mM/2 mM; black trace) or succinate and rotenone (10 mM/1 μM; grey trace) were treated with oligomycin A (2.5 μg ml^−1^) and FCCP (0.5 μM titration) to assess leak respiration and spare respiratory capacity respectively. (**c**) RCR (respiratory control ratio, calculated as the ratio of State 3/State 4 respiration) for freshly isolated mitochondria and trehalose freeze-thawed mitochondria from the same preparation energised using glutamate/malate (10 mM/2 mM). Statistical significance was calculated using a one-way ANOVA corrected for multiple comparisons using Tukey method (NS *P* > 0.05; GraphPad Prism). (**d**) RCR for 3 month cryopreserved mitochondria energised using glutamate/malate (10 mM/2 mM). (**e**) Mitochondria (1 mg protein ml^−1^) were incubated in the presence of 1 mM ADP and either glutamate/malate (10 mM/2 mM) or succinate/rotenone (10 mM/1 μM) and total ATP content was measured after 45 minutes. Luminescence was measured in the presence and absence of respiratory inhibitors antimycin A (2.5 μM) and oligomycin A (2.5 μg ml^−1^) to determine background. Data are expressed as means (±s.d.) of at least three independent experiments using mitochondria that had been cryopreserved for between 1 and 3 months. Abbreviations: Mito; mitochondria, Glu; glutamate, Mal; malate, Succ; succinate, FCCP; carbonyl cyanide-p-trifluoromethoxyphenylhydrazone.

In light of the suggestion of respiratory compromise described previously in trehalose-preserved freeze-thawed mitochondria^38^, mitochondrial functionality needed to be confirmed by assessing key bioenergetic parameters. Rates of mitochondrial oxygen consumption (black trace) and dissolved oxygen (grey trace) were analysed using closed-chamber high resolution respirometry (Fig. 1a). State 4 respiration was observed in the presence of complex I-linked respiratory substrates (glutamate plus malate) (Fig. 1a,b). Adenosine diphosphate (ADP) phosphorylation at the F_1_F_O_-ATP synthase stimulated a respiratory burst characteristic of State 3 respiration (Fig. 1a,b). Sequential addition of the nicotinamide adenine dinucleotide (NADH): ubiquinone oxidoreductase inhibitor rotenone and succinate stimulated complex II-driven State 3 respiration (Fig. 1a). Succinate feeds electrons directly to the ubiquinone (UQ) pool and fewer protons are translocated per electron pair compared to NADH-linked substrates, therefore a higher rate of respiration is required to produce the same proton cycling rate^39^. Non-phosphorylating leak respiration was observed after F_1_F_O_-ATP synthase inhibition using oligomycin A, and maximal electron transport flux determined after titrated membrane uncoupling using carbonyl cyanide-p-trifluoromethoxyphenylhydrazone (FCCP; Fig. 1b). To determine any loss of function following freeze-thaw, freshly isolated mitochondria were compared to those freshly cryopreserved from the same mitochondrial preparation. A respiratory control ratio (RCR; calculated as the ratio of State 3 to State 4 respiration) of greater than 5 was observed for both conditions and no significant difference in RCR was observed following cryopreservation (Fig. 1c). Additionally, calculation of the RCR for a different batch of mitochondria cryopreserved for 3 month also suggested that good functionality had been retained throughout storage (Fig. 1d).

Proton flux from the inter-membrane space (IMS), through the F_1_F_O_-ATP synthase is coupled to the phosphorylation of ADP to adenosine triphosphate (ATP) in the matrix. Rates of ATP synthesis were assessed using a luciferase catalysed reaction, where ATP is utilised as co-substrate in the oxidation of luciferin. The enzymatic activity of luciferase is therefore proportional to ATP concentration in the solution. Freeze-thawed mitochondria were incubated in the presence of ADP and complex I- or complex II-linked respiratory substrates (Fig. 1e). Antimycin A and oligomycin A were used to inhibit ATP synthesis and determine background luminescence. Figure 1e demonstrates energised freeze-thawed mitochondria are capable of synthesising ATP and respond appropriately to inhibition.

Collectively, our data suggest that cryopreserved mitochondria retain respiratory function, remain capable of complex I and complex II driven respiration, have spare respiratory capacity and are capable of ATP synthesis.

### Validation of cryopreserved mitochondria by assessment of mitochondrial membrane potential

**Figure 2:**
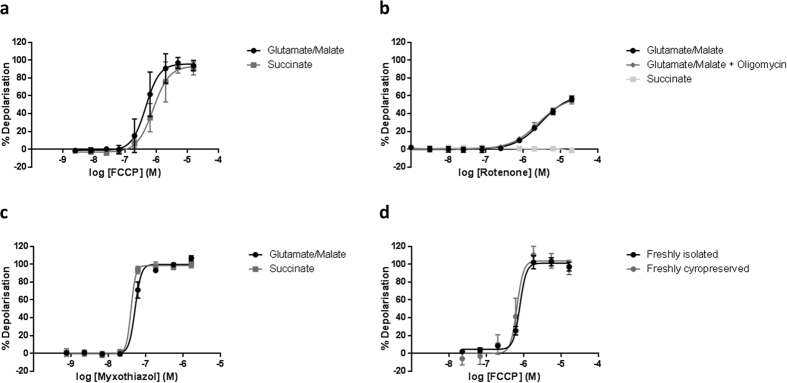
Validation of freeze-thawed mitochondria using TMRM. Rat liver mitochondria (1 mg protein ml^−1^) were incubated with TMRM (2 μM) in the presence of respiratory substrates and compounds: (**a**) FCCP; (**b**) rotenone ± oligomycin A (2.5 μg ml^−1^) and (**c**) myxothiazol. Mitochondria were exposed to mitochondrial toxins in a 3-fold dilution series and TMRM fluorescence (ex. 560/em. 590 nm) measured after 10 minute incubation. (**d**) FCCP concentration-response in freshly isolated mitochondria and trehalose freeze-thawed mitochondria from the same preparation. Results are expressed as percentage (%) depolarisation, normalised to DMSO (0% depolarisation) and antimycin A (2.5 μM) plus oligomycin A (2.5 μg ml^−1^; 100% depolarisation). Data are expressed as means (±s.d.) of at least three independent experiments using mitochondria that had been cryopreserved for between 1 and 3 months. Curve fitting used a 4-parameter logistic equation (GraphPad Prism). Abbreviations: FCCP; carbonyl cyanide-p-trifluoromethoxyphenylhydrazone.

Under steady-state conditions, the extrusion of protons from the mitochondrial matrix into the IMS is balanced with proton re-entry via the F_1_F_O_-ATP synthase or through proton leak^39^. The resulting proton current in energised mitochondria produces an electrochemical gradient (proton motive force; pmf), composed of two components: a membrane potential (ΔΨ_m_) and pH gradient (ΔpH) gradient. Mitochondrial membrane potential can be assessed using the voltage-sensitive cationic lipophilic probe, tetramethylrhodamine methylester (TMRM), which partitions and accumulates in the mitochondrial matrix. When TMRM is loaded at high concentration, fluorescence within the mitochondria is auto-quenched. Any disruption to the mitochondrial membrane potential (i.e. membrane uncoupling or electron transport inhibition) depolarises the membrane and consequent TMRM redistribution yields an increase in fluorescence as auto-quenching is relieved^40^. Under these conditions, fluorescence intensity is a non-linear function of dye concentration. Increasing concentrations of FCCP, myxothiazol, and to a lesser extent rotenone, depolarised glutamate/malate-energised mitochondria (Fig. 2a–c). Addition of oligomycin A yielded no effect on rotenone-induced depolarisation, suggesting residual ATP is not responsible for the incomplete loss of transmembrane potential (Fig. 2b). Instead, the likely explanation for the incomplete depolarisation using rotenone is the generation and supply of Krebs cycle intermediates entering the electron transport chain beyond complex I. Mitochondrial depolarisation was also observed in a dose-dependent fashion in the presence of FCCP and myxothiazol in complex II-driven respiration using succinate (Fig. 2a,c). Rotenone failed to depolarise mitochondria, as succinate bypasses the quinone binding site of complex I (Fig. 2b). Additionally, no difference in FCCP-mediated depolarisation was observed between freshly isolated mitochondria and mitochondria from the same preparation that had undergone a cryopreservation/thaw cycle (Fig. 2d).

Together these data suggest, freeze-thawed mitochondria are capable of generating a membrane potential through electron entry via complex I and complex II and, also, respond as expected to well-described mitochondrial toxins.

### Identification of ER-000444793 by high-throughput screening in cryopreserved mitochondria

Ca^2+^-induced mitochondrial swelling is a well-established method to determine propensity to mPT. In healthy, polarised mitochondria, the inner mitochondrial membrane (IMM) provides a highly selective barrier to ions and solutes. Mitochondrial permeability transition results in loss of membrane integrity, leading to equilibration of solutes and osmotic mitochondrial swelling. Swollen mitochondria transmit more light, and so compound activity can be determined in a high-throughput manner simply by quantifying absorbance^7^,^41^,^42^.

**Figure 3:**
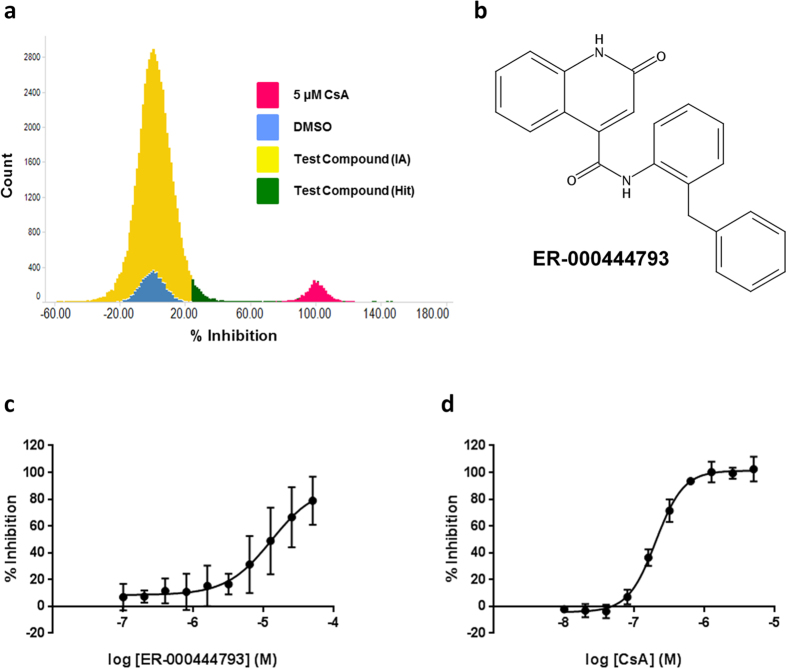
Identification of ER-000444793 from a high throughput screen using freeze-thawed mitochondria. Rat liver mitochondria (2 mg protein ml^−1^), energised using succinate (10 mM) and rotenone (1 μM) were exposed to a CaCl_2_ bolus (150 μM). Absorbance (540 nm) was measured after 20 minutes. (**a**) Graphical representation of the metrics from the 50,000 compound screen identifying the ‘hit’ compound cut-off >20% inhibition. (**b,c**) ER-000444793; N-(2-benzylphenyl)-2-oxo-1H-quinoline-4-carboxamide, was identified from the screen and dose-dependently inhibits Ca^2+^ induced mitochondrial swelling. (**d**) Using the same mitochondrial preparations, CsA also delayed pore opening in response to the Ca^2+^ bolus. Results are expressed as % inhibition, normalised to DMSO (0% inhibition) and CsA (5 μM; 100% inhibition). Data are expressed as means (±s.d.) of at least three independent experiments. Curve fitting used a 4-parameter logistic equation (GraphPad Prism). Abbreviations: IA; inactive, CsA; cyclosporin A.

A 50,000 compound screen was performed using the Ca^2+^-induced mitochondrial swelling assay. The use of cryopreserved mitochondria allowed compound screening to be completed in less than 8 working days. Mitochondria were energised by succinate, and rotenone was included to prevent both accumulation of oxaloacetate, a potent succinate dehydrogenase (SDH) inhibitor and reversal of electron flow to complex I. Compounds were assayed at a single compound concentration and a total of 475 compounds were identified as producing greater than 20% inhibition (0.95% hit rate) (Fig. 3a). Those compounds producing greater than 20% inhibition were re-tested (n = 3) for confirmatory effects or not. In total, 163 compounds were then taken through to IC_50_ determination studies, from which ER-000444793 (Fig. 3b) was identified as producing an IC_50_ of 12.9 μM (Fig. 3c). CsA was used as a positive control producing an IC_50_ of 0.2 μM (Fig. 3d). An average Z′ of 0.58 was achieved throughout screening, satisfying our requirements for consistency and quality of the assay^43^.

### ER-000444793 delays mitochondrial membrane potential depolarisation in response to Ca^2+^ overload

**Figure 4:**
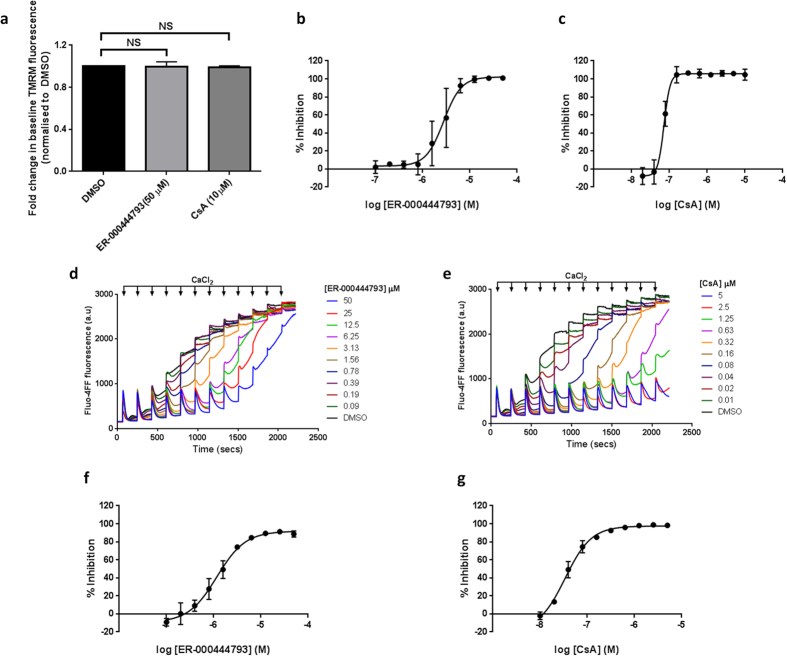
Response of freeze-thawed mitochondria to Ca^2+^-induced mPT. Rat liver mitochondria (2 mg protein ml^−1^) were incubated with TMRM (5 μM) in the presence of succinate (10 mM) and rotenone (1 μM). (**a**) TMRM fluorescence pre-read (ex. 560/em. 590 nm) revealed no significant effect on ER-000444793 (50 μM) or CsA (10 μM) after 10 minutes incubation. Statistical significance was calculated using a one-way ANOVA corrected for multiple comparisons using Tukey method (NS *P* > 0.05; GraphPad Prism). Mitochondria were incubated with (**b**) ER-000444793 or (**c**) CsA in a 2-fold dilution series for 10 minutes prior to the addition of a CaCl_2_ bolus (150 μM). TMRM fluorescence was measured after 20 minutes incubation. Results are expressed as % depolarisation, normalised to DMSO (0% inhibition) and CsA (5 μM; 100% inhibition). (**d–g**) Rat liver mitochondria (1 mg protein ml^−1^) were incubated with Fluo-4FF (0.35 μM) in the presence of succinate (10 mM) and rotenone (1 μM). Pulses of CaCl_2_ (10 μM) were added sequentially and extra-mitochondrial fluorescence measured. Representative raw traces of Ca^2+^ uptake in the presence of (**d**) ER-000444793 and (**e**) CsA demonstrate complete Ca^2+^ uptake prior to concentration-dependent, Ca^2+^-induced mPT. (**f,g**) Area under the curve was calculated between 240–840 seconds and results are expressed as % inhibition, normalised to DMSO (0% inhibition) and CsA (5 μM; 100% inhibition). Data are expressed as means (±s.d.) of at least three independent experiments. Curves fitting used a 4-parameter logistic equation (GraphPad Prism). Raw traces are representative of at least three independent experiments. Abbreviations: NS; not significant, CsA; cyclosporin A, TMRM; tetramethylrhodamine methylester. a.u; arbitrary unit.

The mitochondrial swelling assay provides a useful measure of a compound’s effect on delaying permeability transition. However, it can lead to the identification of false positives. If using the swelling assay alone, mitochondrial depolarising agents, inhibitors of the mitochondrial Ca^2+^ uniporter (MCU) or phosphate carrier would present as false positive hits as they will effect mitochondrial electrophoretic Ca^2+^ uptake and Ca^2+^ sequestration, thereby preventing Ca^2+^-induced mPT. Therefore, to eliminate false positives, a second approach was used. Ca^2+^-induced mPTP opening is also associated with loss of selective permeability and depolarisation of mitochondrial inner membrane, and the latter effect can readily be measured using the voltage sensitive cationic probe TMRM. ER-000444793 was confirmed to have no significant effect on baseline TMRM fluorescence (Fig. 4a; *P* > 0.05) but delayed mitochondrial membrane potential depolarisation in a dose-dependent manner after the addition of a CaCl_2_ bolus (Fig. 4b). ER-000444793 inhibited mPT with an IC_50_ of 2.8 μM as measured using this assay, compared to 72 nM with the positive control, CsA (Fig. 4c).

### ER-000444793 improves mitochondrial Ca^2+^ retention capacity

Membrane potential-dependent Ca^2+^ accumulation through the MCU shapes cellular Ca^2+^ dynamics and homeostasis^44^,^45^,^46^,^47^. Under extremes of cellular Ca^2+^, mitochondrial buffering capacity is exceeded and the mPTP opens. Ca^2+^ retention capacity of isolated mitochondria can be measured through the addition of repeated small pulses of CaCl_2_ to a suspension of mitochondria, providing an exquisitely sensitive measure of propensity to mPT^48^.

Trehalose-preserved, freeze-thawed rat liver mitochondria are capable of Ca^2+^ uptake, as extramitochondrial Fluo-4FF fluorescence returned to baseline following a single CaCl_2_ addition (Fig. 4d,e - DMSO). Neither ER-000444793 nor CsA exhibited effects on Ca^2+^ uptake after the first Ca^2+^ injection, suggesting absence of effects on mitochondrial Ca^2+^ uptake or membrane potential (Fig. 4d,e). Sensitivity to Ca^2+^-induced mPTP opening was identified as failure of mitochondria to remove added Ca^2+^ from the solution and release of internalised stores. ER-000444793 improved mitochondrial Ca^2+^ tolerance with an IC_50_ of 1.2 μM using this assay (Fig. 4d,f). CsA was used as a positive control, improving mitochondrial Ca^2+^ tolerance with an IC_50_ of 34 nM (Fig. 4e,g). A summary of three screening assays used to identify and characterise ER-000444793 can be seen in Table 2. Taken together, these data show ER-000444793 potently and dose-dependently inhibits Ca^2+^-induced mPT.

### Pharmacology of ER-000444793 is comparable in mitochondria isolated from human cell lines

**Figure 5:**
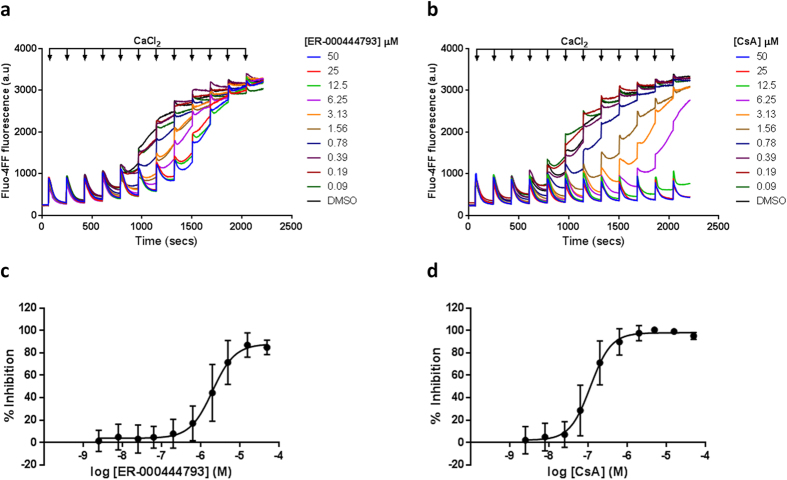
Ca^2+^ retention capacity of freeze-thawed Hela S3 human mitochondria. Hela S3 mitochondria (0.5 mg protein ml^−1^) were incubated with Fluo-4FF (0.35 μM) in the presence of succinate (10 mM) and rotenone (1 μM). Pulses of 5 μM CaCl_2_ were sequentially added at 3 minute intervals and extra-mitochondrial Fluo-4FF fluorescence measured. Representative raw traces of Ca^2+^ uptake in the presence of (**a**) ER-000444793 and (**b**) CsA demonstrate complete Ca^2+^ uptake prior to concentration-dependent Ca^2+^-induced mPT. (**c,d**) Area under the curve was calculated between 60–660 seconds and results are expressed as % inhibition, normalised to DMSO (0% inhibition) and CsA (5 μM; 100% inhibition). Data are expressed as means (± s.d.) of at least three independent experiments. Curve fitting used a 4-parameter logistic equation (GraphPad Prism). Raw traces are representative of at least three independent experiments. Abbreviations: CsA; cyclosporin A, a.u; arbitrary unit.

mPT has been demonstrated across many species, including mitochondria from human cells^49^, rodents^21^, fish^50^ and yeast^51^. To confirm the screening data and demonstrate consistent compound pharmacology in human samples, mitochondria were isolated from HeLa S3 cells and Ca^2+^ retention capacity assessed. HeLa S3 mitochondria were isolated by a method that paralleled the isolation and trehalose preservation of mitochondria isolated from rat liver. Mitochondria were energised using succinate and rotenone and subject to small pulses of CaCl_2_ in the presence of an extra-mitochondrial Ca^2+^ sensitive fluorescent dye. As observed with rat liver mitochondria, trehalose-preserved, freeze-thawed Hela S3 mitochondria are capable of Ca^2+^ uptake (Fig. 5a,b – DMSO). HeLa S3 mitochondria were responsive to ER-000444793 treatment and mitochondrial Ca^2+^ retention capacity dose-dependently increased, delaying the onset of mPT with an IC_50_ of 2.0 μM (Fig. 5c). Cyclosporin A was used as a positive control and improved mitochondrial Ca^2+^ tolerance with an IC_50_ of 120 nM (Fig. 5d). Together, these data suggest that ER-000444793 is a potent inhibitor of mPT and pharmacology is comparable between rodents and man (shown in Table 3).

### Inhibition mPT by ER-000444793 is independent of cyclophilin D

As the molecular identity of the mPTP remains uncertain, to date, much of the focus with respect to pharmacological modulation of the mPTP has been centred on CypD^24^,^30^. Genetic ablation and chemical inhibition of CypD have both been demonstrated to delay mPT in isolated mitochondria and cells^21^,^23^,^24^. However, off-target inhibition of cyclophilin family members, notably immunosuppression related to the ternary CsA-CypA-calcineurin complex, remains problematic and hence there is need for more targeted therapies^35^.

**Figure 6:**
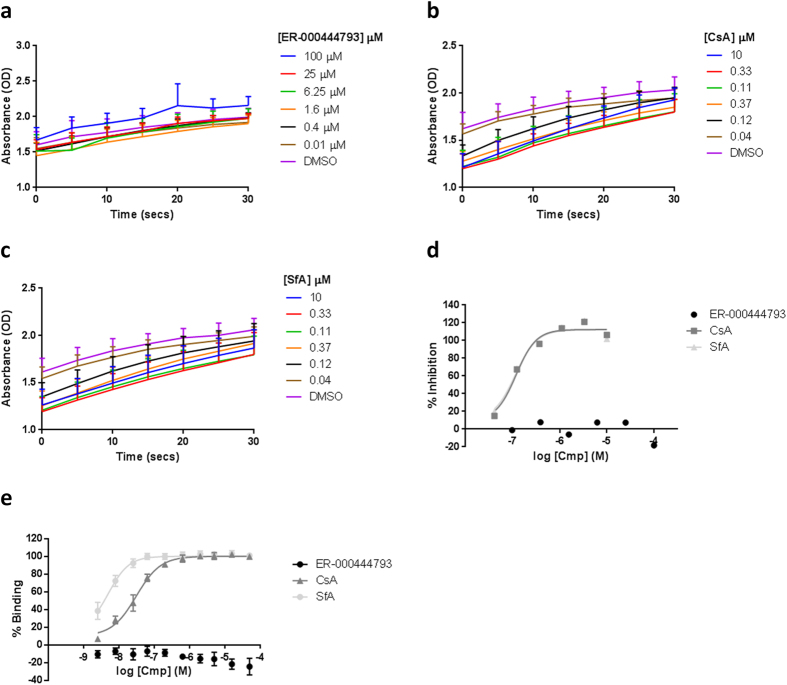
Assessment of CypD functional activity and compound binding. rhCypD protein was incubated with chymotrypsin and compound for 30 minutes. N-Succinyl-Ala-Ala-Pro-Phe-*p*-nitroanilide was added and absorbance (405 nm) measured for 60 seconds at 3 second intervals. (**a**) Kinetic traces in the presence of ER-000444793 demonstrated no inhibition of enzyme activity. (**b,c**) Dose-dependent inhibition of rhCypD enzymatic activity observed with CsA and SfA. (**d**) Area-under-the-curve from a-c was calculated from the first 15 seconds of the reaction and results expressed as % inhibition, normalised to DMSO (0% inhibition) and CsA (5 μM; 100% inhibition). (**e**) rhCypD TR-FRET assay demonstrates a dose-dependent decrease in FRET by both CsA and SfA but no effect of ER-000444793. Data are expressed as % binding, normalised to DMSO (0% binding) and CsA (5 μM; 100% binding). All data are expressed as means (±s.d.) of at least three independent experiments. Abbreviations: CsA; cyclosporin A, SfA; sanglifehrin A, O.D; optical density, Cmp; compound.

To establish whether ER-000444793 exerts its effects through modulating CypD enzymatic activity, its ability to inhibit PPIase activity of recombinant human CypD (rhCypD) protein was measured. Cyclophilins belong to a group of protein which possess peptidyl prolyl cis-trans isomerase (PPIase) activity and catalyse a change in stereochemistry around proline residues in target proteins. PPIase activity of CypD is necessary for the positive regulation of pore opening^21^. *In vitro* PPIase reaction is extremely rapid, however using appropriate kinetics, both CsA (Fig. 6b) and SfA (Fig. 6c) were observed to dose-dependently inhibit CypD PPIase activity. In contrast, ER-000444793 had no effect on CypD enzymatic activity up to a concentration of 100 μM (Fig. 6a). Inhibition values calculated using area-under-the-curve revealed potent inhibition with CsA and SfA (IC_50_  = 106 nM and IC_50_ = 99 nM respectively) (Fig. 6d).

To confirm the functional data and investigate whether ER-000444793 binds CypD, a CsA/CypD homogenous time-resolved fluorescence (HTRF) assay was used to study compound binding. Both CsA and SfA dose-dependently decreased HTRF signal, suggesting displacement of labelled CsA from rhCypD protein (CsA IC_50_  = 23 nM and SfA IC_50_ = 5 nM). In contrast, ER-000444793 had no effect up to a concentration of 50 μM, indicating lack of displacement of labelled-CsA from rhCypD protein (Fig. 6e). Together, these data suggest the mechanism of ER-000444793 in delaying mPT is independent of CypD functional inhibition.

### ER-000444793 does not affect mitochondrial or cellular health

**Figure 7:**
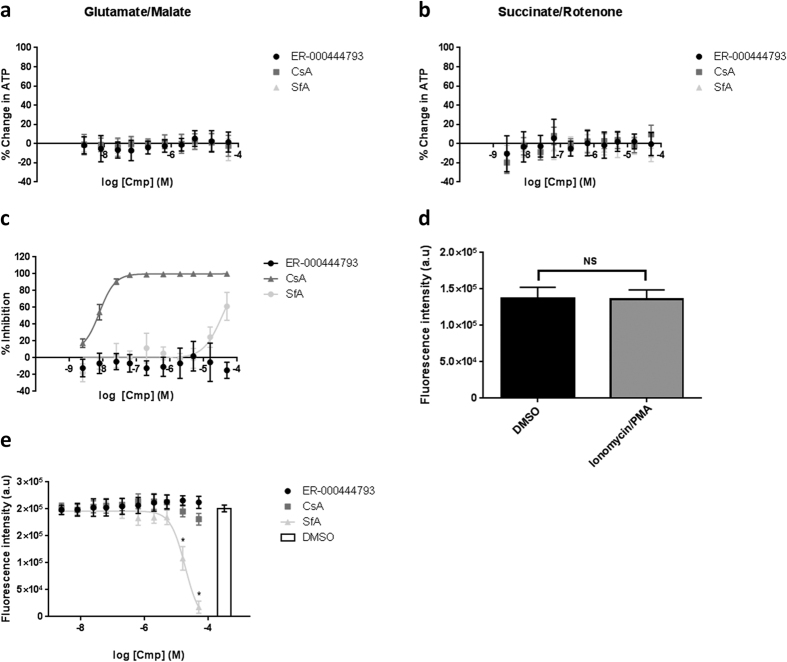
Assessment of both mitochondrial and cellular toxicity. (**a,b**) Rat liver mitochondria (1 mg protein ml^−1^) were incubated with compound for 10 minutes in the presence of either glutamate (10 mM) and malate (2 mM) or succinate (10 mM) and rotenone (1 μM). ADP (5 mM) was added and incubated for 45 minutes. Mitochondria were lysed and total ATP quantified by luminescence using CellTiter Glo reagent (Promega, Madison, WI). No effect of either ER-000444793, CsA or SfA was observed under either respiratory substrate condition. Results are expressed as % inhibition of ATP, synthesis normalised to DMSO (0% inhibition) and antimycin A (2.5 μM) plus oligomycin A (2.5 μg ml^−1^; 100% inhibition). Data are expressed as means (± s.d.) of at least three independent experiments. (**c**) Assessment of immunosuppression and cellular toxicity in Jurkat NFAT-reporter cells. Jurkat cells were incubated with compound for 45 minutes prior to the addition of ionomycin (0.5 μM) and PMA (50 nM). NFAT reporter activity was assessed after 20 hours treatment with either ER-000444793, CsA or SfA and quantified by luminescence using Bright Glo reagent (Promega, Madison, WI). Data are expressed as % inhibition, normalised to DMSO (0% inhibition) and CsA (5 μM; 100% inhibition). (**d**) Toxicity of ionomycin/PMA treatment was assessed in Jurkat cells using Alamar blue after 20 hours incubation. No significant effect was observed with treatment. Data are expressed as fluorescence intensity and statistical significance calculated using paired two-tailed student’s *t*-test using GraphPad Prism (NS *P* > 0.05). (**e**) Jurkat cells were incubated with compound for 20 hours and toxicity assessed using Alamar Blue. Data are expressed as fluorescence intensity and compared using multiple t-tests comparing treatments to DMSO, corrected for multiple comparisons using Holm-Sidak method*, P* < 0.05 defined as being statistically significant. All data are expressed as means (± s.d.) of three independent experiments. Abbreviations: CsA; cyclosporin A, SfA; sanglifehrin A, Cmp; compound, O.D; optical density, NS; not significant, PMA; phorbol 12-myristate.

To assess any mitochondrial toxicity of ER-000444793, rates of mitochondrial ATP synthesis were measured using complex I-linked (glutamate and malate) or complex II-linked (succinate) substrates. ER-000444793 had no effect on mitochondrial ATP synthesis up to a concentration of 50 μM (Fig. 7a,b). In addition, CsA and SfA also had no effect on total ATP levels (Fig. 7a,b), suggesting all compounds up to 50 μM are free of mitochondrial toxicity.

Due to inherent isoform homology between cyclophilins, use of CsA and SfA for treating mitochondrial dysfunction is limited by their non-isoform selectivity. Notably, CsA bridges the CypA-calcineurin ternary complex resulting in immunosuppression^27^. To identify any possible immunosuppressive activity of ER-000444793, Jurkat cells stably expressing an NFAT-luciferase reporter were utilised. Activation of NFAT transcriptional signalling was achieved using ionomycin and phorbol 12-myristate (PMA), generating a robust upregulation of luciferase gene expression. ER-000444793 revealed no effect on luciferase expression up to 50 μM compared to controls (Fig. 7c). In contrast the prototypical immunosuppressive agent CsA revealed a dose-dependent decrease on NFAT-reporter activity, while SfA had no effect up to a concentration of ~5 μM (Fig. 7c). Alamar Blue was used to assess cell viability as it relies on the conversion of resazurin in metabolically active cells to the fluorescent molecule resorufin. The Alamar Blue assay revealed no significant toxicity after PMA and ionomycin alone (Fig. 7d; *P* > 0.05). By this assay, ER-000444793 and CsA are free of cellular toxicity and only SfA at concentrations above ~5 μM significantly affected cell health, identifying cell toxicity as the mechanism behind apparent NFAT-reporter activity (Fig. 7e). Together, these data confirm that ER-000444793 is non-toxic, having no mitochondrial or cellular liabilities at concentrations tested and, similarly, that it does not inhibit NFAT signalling.

## Discussion

In this work, we have exploited a method to isolate mitochondria, preserving function after freeze-thaw in the presence of trehalose^38^. We successfully optimised and deployed this method to generate functional mitochondrial preparations from both rat tissue and human cells. Bioenergetic evaluation of cryopreserved mitochondria revealed their quality to be comparable to those freshly isolated. Mitochondrial isolation was substantially scaled-up, such that large quantities of mitochondria were produced and batch frozen. Functional activity of batch-prepared, freeze-thawed isolated mitochondria was assessed using the Ca^2+^-induced mitochondrial swelling assay. Only 3 preparations from a total of 20 batches failed quality control, based on assay robustness and sensitivity, suggesting batch-to-batch variability is minimal and bulk preparation has little effect on assay and mitochondrial quality. Despite prolonged periods at −80 °C, mitochondria maintained function over time, as assay quality (defined by the Z′ factor) in the Ca^2+^ retention assay, routinely used for compound analogue screening, remained high.

Despite the reported modest respiratory limitations of freeze-thawed mitochondria^38^, our preparations of trehalose-preserved mitochondria were capable of maintaining respiration and demonstrate appropriate respiratory coupling, maintenance of a transmembrane potential and ATP synthesis. These intrinsic mitochondrial functions are essential for mitochondrial Ca^2+^-uptake and crucial to be able to assess mPT *in vitro*. The ability to maintain mitochondrial function after freeze-thaw using trehalose therefore provides an approach to perform rapid, large primary and confirmatory secondary, compound screens, necessary in most drug discovery campaigns.

We took advantage of our optimised mitochondrial preservation methodology and performed a HTS of 50,000 compounds using a Ca^2+^-induced mitochondrial swelling assay. Batch preparation and maintenance of mitochondrial function following cryopreservation allowed primary compound screening to be completed in less than 8 working days. Results from the HTS identified over 150 compounds from multiple distinct chemical series inhibiting mPT. ER-000444793, a low molecular weight compound, was taken forward for further study, based on its ability to potently inhibit mPT and its chemical structure. Activity of ER-000444793 was demonstrated after observing positive effects in ability to delay Ca^2+^-induced IMM depolarisation and increased mitochondrial Ca^2+^ retention capacity. ER-000444793 also failed to affect resting membrane potential or Ca^2+^ uptake in isolated mitochondria, suggesting pharmacological effects are limited to inhibition of mPT. Pharmacology of ER-000444793 was also confirmed in a Ca^2+^ retention assay using mitochondria isolated from HeLa S3 cells, preserved using the same trehalose methodology, suggesting a conserved mode of action between rodents and humans.

To date, only CypD has been truly agreed by numerous independent laboratories as being a critical regulator of pore opening, with substantial genetic and pharmacological studies having identified the role of CypD in potentiating pore opening^21^,^25^,^49^. Most mPTP inhibitors reported in the literature, such as NIM-811, CsA and SfA desensitize the pore to Ca^2+^-induced opening, through inhibition of CypD^24^,^30^,^52^. Importantly, therapeutic efficacy of these compounds may be limited as they fail to inhibit pore opening completely as the mitochondrial Ca^2+^ load can still reach the threshold for mPT, as similarly seen after genetic ablation of CypD^21^,^25^. In contrast, ER-000444793 inhibits mPT through a mechanism independent of CypD inhibition. Future work will aim to increase potency and understand the mechanisms involved that mediate the inhibitory activity.

More recently, a number of groups have also undertaken large compound library screens aimed at identifying novel inhibitors of mPTP opening^34^,^36^,^53^. Following structure-activity relationship studies around the *N*-phenyl-benzamide scaffold, Roy *et al*. identified 3-(benzyloxy)-5-chloro-*N*-(4-(piperidin-1-ylmethyl)phenyl)benzamide as providing protection against both Ca^2+^ and oxidative stress-induced mPTP opening^53^. Unfortunately, due to toxicity the therapeutic potential of *N*-phenyl-benzamides in disease models was not assessed. Additionally, a potent cinnamic anilide has been observed to inhibit mPTP opening in response to Ca^2+^ overload, oxidative stress, and activation by chemical cross-linkers^36^. Interestingly, a variant cinnamic anilide was able to cross the blood-brain barrier giving potential utility in neurodegenerative disorders where dysfunctional mitochondria have been observed to play a role, such as amyotrophic lateral sclerosis (ALS)^37^. The cinnamic anilide, GNX-4728 slowed disease progression in a mouse model of ALS, producing a 2-fold increase in lifespan^37^. Much like ER-000444793, a final class of compounds has been identified which inhibited mPT across species with no effect on ATP synthesis and mitochondrial function. Isoxazoles inhibited mPTP opening with potency similar to CsA and improved motor function and muscle structure in zebrafish model of collagen VI congenital muscular dystrophy^34^.

Interestingly, as also observed with ER-000444793, *N*-phenyl-benzamides, cinnamic anilides and isoxazoles inhibit mPT through a mechanism distinct from CypD, avoiding potential limitations associated with off-target effects on multiple cyclophilins and therapeutic efficacy. Together, these compounds represent a novel and diverse collection of tools, which, through further development, may provide clinically utility, as well as insight into the regulatory signalling pathways and molecular architecture of the mPTP complex. Although the structure of the mPTP remains debated, much evidence indicates the F_1_F_O_-ATP synthase as a likely candidate^54^,^55^,^56^,^57^,^58^. Pathogenic F_1_F_O_-ATP synthase dimerisation or exposure of an uncoupling channel within the C-ring have both been proposed to mediate mPT^54^,^56^. ER-000444793 failed to affect ATP synthesis up to a concentration of 50 μM, suggesting, reassuringly, the absence of any effect on the catalytic activity of the complex. The F_1_F_O_-ATP synthase is however, a remarkably complex structure and is likely to have numerous binding sites distinct from regions that affect functional activity. Given the CypD-independent mechanism of ER-000444793, direct effects on F_1_F_O_-ATP synthase complex stability or inhibition of dimerisation remain potential hypotheses as to its mechanism of action.

Cyclophilin A (CypA) is a multifunctional protein and its expression correlates with poor outcome in patients with inflammatory disease^59^. Clinically, CsA is an effective immunosuppressant as it bridges a ternary complex between CypA and calcineurin, inhibiting NFAT signalling^27^. Interestingly, NIM-811, an analogue of CsA, also binds and inhibits distinct cyclophilins, however, unlike CsA, fails to complex CypA-calcineurin, and therefore has no immunosuppressant activity^30^. Using an NFAT-luciferase reporter system, ER-000444793 exhibited no immunosuppressive activity and cellular and mitochondrial toxicities were absent at concentrations up to 50 μM.

Significant literature on the use of mPTP inhibitors has focussed on ischaemia-reperfusion injury of the heart (for review see ref. 60). Oxygen deprivation during ischaemia causes cessation of mitochondrial ATP synthesis and concentrations of adenosine monophosphate (AMP) and inorganic phosphate to rise. As metabolic substrates are diverted away from mitochondria, metabolic acidosis ensues. Removal of excess protons through the sodium/hydrogen exchanger (NHE-1), inactivity of the sodium/potassium ATPase (Na^+^/K^+^-ATPase) and sodium/Ca^2+^ (Na^+^/Ca^2+^) exchange leads to high cytosolic Ca^2+^. Under these conditions, the mPTP remains closed due to low pH^11^. Upon reperfusion, pH-dependent inhibition is relieved, mitochondrial membrane potential is restored and the proceeding overload of mitochondrial Ca^2+^, combined with excessive reactive oxygen species (ROS) and adenine nucleotide depletion create a perfect environment for pore opening^11^. Clinically, conditions where coronary blood flow is interrupted, such as primary percutaneous coronary intervention (PCI) and coronary artery bypass graft (CABG) surgery create an environment primed for mPTP opening and may benefit from the administration of a mPTP inhibitor prior to re-perfusion. The need for alternative inhibitors of mPT was recently demonstrated after CsA failed to improve clinical outcomes above placebo over a year in patients with anterior ST-elevation myocardial infarction (STEMI) who had been referred for primary PCI^61^.

In summary, we validated a method to prepare at scale, high quality mitochondria which maintain robust and reproducible function and assay performance after freeze-thaw over extended periods of storage at −80 °C. The protocols described may prove useful for other future large-scale functional screens using isolated mitochondria. mPTP opening has been implicated in cellular life/death outcomes, making the therapeutic targeting of the pore an appealing strategy in diseases where cytoprotection is desired. Strategies described here have successfully identified a novel and CypD-independent inhibitor of mPT. Structure activity relationship (SAR) analysis using ER-000444793 as a template may provide a basis for future development. Improvements in ER-000444793 potency may ultimately yield therapeutic benefit. We also foresee ER-000444793 as providing a useful biochemical tool to study CypD-independent mPT. Identification of ER-000444793 binding partners and/or mechanism of action may reveal novel determinants and regulators of mPTP opening that are chemically tractable. We anticipate the screening flow and compound described could be useful for advancing the search for novel agents targeting mitochondrial permeability transition and ultimately provide therapeutic benefit for numerous difficult to treat currently intractable diseases and disorders.

## Methods

### Materials

All chemicals and compounds were purchased from Sigma-Aldrich (St. Louis, MO) unless otherwise specified. N-(2-benzylphenyl)-2-oxo-1 H-quinoline-4-carboxamide (ER-000444793; CAS number: 792957-74-5) was purchased from Enamine (Kiev, Ukraine); sanglifehrin A (SfA) was isolated from a fermentation broth; functionalised CsA-NH_2_ was synthesised in-house. Fluorescent probes (tetramethylrhodamine methyl ester perchlorate, TMRM, Fluo-4FF, Alamar Blue) were purchased from Life Technologies (Eugene, OR).

### Cell culture

All cell culture media and supplements were purchased from Life Technologies (Eugene, OR). The human cell line Hela S3 was purchased from ATCC (Manassas, VA) and maintained in Dulbecco’s Modified Eagle Medium (DMEM), high glucose plus GlutaMAX, containing 10% FCS (Atlas Biologicals, Fort Collins, CO). NFAT-RE-luc2 Jurkat cells (Promega, Madison, WI) were cultured in RPMI-1640 containing 10% FCS, MEM non-essential amino acids (1x), sodium pyruvate (1 mM) and hygromycin B (200 μg ml^−1^). All cell lines were maintained at 37 °C under 5% CO_2_.

### Animals

Female Sprague Dawley rats (250–300 g) were purchased from Charles River (Wilmington, MA) and allowed to acclimatise to conditions for four days. Animals were euthanised by cervical dislocation and livers immediately removed. Animal care and procedures were performed in accordance with UK Animals (Scientific Procedures) Act, 1986. Procedures were carried under a UK Home Office licence and studies were approved by Eisai Institutional Animal Care and Use Committee (IACUC).

### Isolation and storage of mitochondria

Mitochondria were isolated in accordance with published protocols^38^, with minor modifications. Fresh rat livers were placed in ice-cold wash buffer (250 mM sucrose, 10 mM KCl, 1 mM EGTA, 1 mM EDTA, 25 mM HEPES, final pH 7.5). Tissue was finely minced with scissors, fat deposits removed and then washed with several changes of ice cold wash buffer. The wash buffer was carefully drained and the remaining tissue was homogenised using 10 strokes of a glass/Teflon potter and drill, set to 1600 rpm, in 5x tissue volume of complete homogenisation buffer (300 mM trehalose, 25 mM HEPES, 1 mM EGTA, 1 mM EDTA, 10 mM potassium chloride, 0.1% essentially fatty acid free bovine serum albumin (BSA) (Sigma, A3803), cOmplete Protease Inhibitor™ (Roche Diagnostics, Mannheim, Germany), final pH 7.5). Homogenates were centrifuged at 800 *g* for 10 minutes at 4 °C; supernatants were then transferred to a clean tube and centrifuged further at 10,300 *g* at 4 °C for 10 minutes. Mitochondrial pellets were washed using complete homogenisation buffer and the final centrifugation step repeated. The pellets were re-suspended in complete homogenisation buffer and the protein concentration was determined by bicinchoninic acid assay (BCA) (Thermo Scientific, Rockford, IL). Mitochondrial suspensions (50 mg protein ml^−1^) were snap-frozen in liquid nitrogen and stored at −80 °C until use.

Mitochondria were isolated from HeLa S3 cells grown on 25 cm × 25 cm tissue culture plates. Cells were scraped in complete homogenisation buffer (as above) and homogenised using 100 strokes of a glass/Teflon potter and drill set to 1600 rpm. Mitochondria were then isolated using the same protocol as described for rat liver, snap-frozen and stored at −80 °C.

All mitochondria were maintained at −80 °C for up to 7 months. Prior to any activity assays, frozen mitochondria were thawed by briefly placing vials in a 37 °C water bath and then kept on ice until required. Unless otherwise stated, assays described were performed using mitochondria isolated from rat liver.

### Validation of frozen mitochondrial activity using TMRM

Rat mitochondria (1 mg protein ml^−1^ final concentration) were incubated in assay buffer (75 mM mannitol, 25 mM sucrose, 5 mM potassium phosphate monobasic, 20 mM Tris base, 100 mM potassium chloride, 0.1% essentially fatty acid free BSA, adjusted to pH 7.4). The buffer was supplemented with either 10 mM L-glutamic acid, monosodium salt; 2 mM L-malic acid sodium salt as electron donor to complex I or 10 mM succinate disodium salt in combination with 1 μM rotenone as electron donor to complex II. Mitochondria were loaded with tetramethylrhodamine methyl ester (TMRM) (2 μM) for 10 minutes at room temperature and mitochondrial suspension (40 μl) was dispensed into each well of a solid bottom black 384 well plate using a Multidrop Combi Reagent Dispenser (Thermo Scientific, Rockford, IL). Increased fluorescence intensity (ex. 540/em. 590 nm) indicated de-quenching of TMRM fluorescence and therefore mitochondrial membrane depolarisation^40^. Fluorescence intensity was determined using Pherastar *FS* (BMG Labtech, Ortenberg, Germany) following 10 minutes compound incubation. Data were analysed using fluorescence intensities, normalised to antimycin A (2.5 μM) oligomycin A (2.5 μg ml^−1^; 100% depolarised) and DMSO (0% depolarised).

### High resolution respirometry

Mitochondrial oxygen consumption was measured using the Oxygraph 2 K (Oroboros Instruments, Innsbruck, Austria). Freeze-thawed mitochondria (0.25 mg protein ml^−1^ final concentration) were re-suspended in mitochondrial respiration medium (3 mM magnesium chloride, 0.5 mM EGTA, 60 mM lactobionic acid, 20 mM taurine, 10 mM potassium phosphate monobasic, 20 mM HEPES, 110 mM sucrose, 0.1% essentially fatty acid free BSA, final pH 7.4) and respiration allowed to stabilise. A sequential titration protocol was followed using a combination of 10 mM L-glutamic acid, monosodium salt, 2 mM L-malic acid sodium salt, 10 mM succinate disodium salt, 1 mM adenosine 5′ diphosphate potassium salt (ADP), 1 μM rotenone, 2.5 μM antimycin A, 0.5 μM carbonyl cyanide p-[trifluoromethoxy]-phenyl-hydrazone (FCCP) and 2.5 μg ml^−1^ oligomycin A at 37 °C under constant stirring (750 rpm) and Oxygraph 2 K calibrated using oxygen solubility factor 0.92.

### ATP synthesis assay

Mitochondria (1 mg protein ml^−1^ final concentration) were re-suspended in ATP synthesis buffer (20 mM Tris base 0.6 M sorbitol, 15 mM potassium phosphate monobasic, 10 mM magnesium sulphate, 2.5 mg ml^−1^ essentially fatty acid free BSA, final pH 7.4) containing either 10 mM L-glutamic acid, monosodium salt; 2 mM L-malic acid sodium salt or 10 mM succinate disodium salt; 1 μM rotenone and mitochondrial suspension (20 μl) was dispensed into a clear bottom black-walled 384 well plate using a Multidrop Combi Reagent Dispenser (Thermo Scientific, Rockford, IL). Mitochondria were incubated in the presence of compound for 10 minutes. Assay buffer (20 μl) containing ADP (5 mM final assay concentration) was added to all wells and incubated at room temperature for 45 mins. The reaction was stopped by the addition of CellTiter-Glo reagent (Promega, Madison, USA) and the plate was left at room temperature for 10 minutes to stabilise. Luminescence was recorded using Pherastar *FS* (BMG Labtech, Ortenberg, Germany). Data were analysed using luminescent intensities, normalised to antimycin A (2.5 μM) in combination with oligomycin A (2.5 μg ml^−1^; 100% inhibition) and DMSO (0% inhibition).

### Ca^2+^-induced mitochondrial swelling

Upon mPTP opening, mitochondria become swollen due to disruptions in ionic and solute balance. Thawed mitochondria were diluted to 4 mg protein ml^−1^ in ice-cold swelling assay buffer (150 mM mannitol, 50 mM sucrose, 5 mM potassium phosphate monobasic, 100 mM potassium chloride, 0.2% essentially fatty acid free BSA, 10 mM succinate disodium salt, 1 μM rotenone, 25 mM HEPES final pH 7.4) and 20 μl of the suspension was dispensed into a clear bottom black-walled 384 well plate containing compound using a Multidrop Combi Reagent Dispenser (Thermo Scientific, Rockford, IL). Plates were incubated at room temperature for 10 minutes during which baseline absorbance was recorded. Assay buffer (20 μl) containing calcium chloride (CaCl_2_; 150 μM final assay concentration) was added to all wells and incubated for 30 minutes at room temperature. Mitochondrial swelling was identified through decreases in absorbance at 540 nm (Pherastar *FS*, BMG Labtech, Ortenberg, Germany) and data normalised to CsA (5 μM; 100% inhibition) and DMSO (0% inhibition).

### Compound screening

For high-throughput screening, a 50,000 sub-set of the Eisai compound collection was used. Single shot analysis was performed at ~13 μg ml^−1^ (small molecules) and ~ 1.5 μg ml^−1^ (natural products). Initial hits were identified using the Ca^2+^-induced swelling assay in freeze-thawed rat mitochondria. All compounds were dispensed using an ECHO 550 (Labcyte, Sunnyvale, CA,) to a final maximal DMSO concentration of 0.5%. To pass our quality control measures, each plate was required to have: a Z′ > 0.35 using 5 μM CsA and DMSO as positive and negative controls respectively; >50% inhibition using 0.5 μM CsA^43^. Absorbance was measured before Ca^2+^ addition to ensure physicochemical compound properties did not interfere with assay readout. Hit compounds were selected based on >20% inhibition after normalisation to controls.

### Ca^2+^-induced mitochondrial membrane depolarisation

Mitochondria were diluted to 2 mg protein ml^−1^ in TMRM assay buffer (10 μM TMRM, 150 mM mannitol, 50 mM sucrose, 5 mM potassium phosphate monobasic, 100 mM potassium chloride, 0.2% BSA, 10 mM succinate disodium salt, 1 μM rotenone, 25 mM HEPES final pH 7.4). TMRM loaded mitochondrial suspension (20 μl) was dispensed into a solid bottom black walled plate containing compound using a Multidrop Combi Reagent Dispenser (Thermo Scientific, Rockford, IL) and incubated for 10 mins at room temperature. CaCl_2_ in assay buffer (20 μl; 75 μM final assay concentration) was added to all wells and incubated for 30 minutes at room temperature. Fluorescence intensity was read on the PheraStar *FS* (ex. 540/em. 590 nm) and data normalised to CsA (5 μM; 100% inhibition) and DMSO (0% inhibition).

### Ca^2+^ retention capacity (CRC) assay

Assessment of Ca^2+^ retention capacity can be used to assess *in vitro* sensitivity to Ca^2+^ of isolated mitochondrial preparations. Mitochondria were washed in ice cold CRC assay buffer (75 mM mannitol, 25 mM sucrose, 5 mM potassium phosphate monobasic, 20 mM Tris base, 100 mM potassium chloride, 0.1% bovine serum albumin, adjusted to pH 7.4) to remove residual EDTA and re-suspended (1 mg protein ml^−1^) in complete CRC assay buffer containing succinate disodium salt (10 mM), rotenone (1 μM) and Fluo-4FF penta-potassium salt (0.35 μM; K_d_ Ca^2+^ in buffer: ~9.7 μM). Mitochondrial suspension (20 μl) was dispensed into a clear bottom black-walled plate containing compound using a Multidrop Combi Reagent Dispenser (Thermo Scientific, Rockford, IL) and incubated for 10 mins at room temperature. Extra-mitochondrial fluorescence (ex. 470–495/em. 515–575) was measured at 3 second intervals (FLIPR^TETRA^, Molecular Devices, Sunnyvale, CA) over 35 minutes at room temperature. CaCl_2_ (10 μM final concentration per addition) in complete assay buffer (2.5 μl additions to 20 μl) was repeatedly added at 3 minute intervals. Data were analysed using the area under the curve calculated between 240–840 seconds and normalised to CsA (5 μM; 100% inhibition) and DMSO (0% inhibition). CRC assay buffer was pre-treated with Chelex 100 resin (Sigma-Aldrich, St. Louis, MO) to remove any contaminating Ca^2+^, and resin removed through filtration.

### Recombinant human CypD (rhCypD) protein production

An expression construct, previously described to determine the crystal structure of CypD^62^ was created with minor modifications. Briefly, cDNA synthesised *de novo* encoding amino acids 44 to 207 of the full length human CypD sequence (Uniprot P30405) without a stop codon was inserted into the Pet21a vector (Novagen) at the Nde1 and BamH1 restriction sites. The resulting C-terminal His-tagged truncated CypD was expressed in BL21(DE3) *Escherichia coli* (Agilent Technologies, Santa Clara, CA) by inducing expression with 1 mM isopropyl β-D-1-thiogalactopyranoside (IPTG) at 37 °C for 2 hours. Cultures were harvested by centrifugation and then re-suspended in 50 mM phosphate buffer pH 7.0, 0.5 M NaCl, 1 mM dithiothreitol (DTT) and cOmplete™ protease inhibitor (Roche Diagnostics, Mannheim, Germany). Cells were lysed by passing through a French Press twice at 1000 psi and the resulting lysate was centrifuged at 14,000 *g* for 20 minutes at 4 °C. His-tagged rhCypD protein was purified from the supernatant using a 1 ml HisTrap FF column (GE Healthcare, Little Chalfont, Buckinghamshire, UK) by fast protein liquid chromatography (FPLC) and then buffer exchanged into phosphate buffered saline (PBS) containing 10% glycerol and 1 mM DTT before storing at −20 °C.

### Recombinant human CypD (rhCypD) peptidyl-prolyl *cis trans* isomerase (PPIase) activity assay

PPIase activity was measured *in vitro* using rhCypD and enzyme linked-confirmation specific cleavage of N-Succinyl-Ala-Ala-Pro-Phe-*p*-nitroanilide to yield a colorimetric product. Ice-cold chymotrypsin (50 μl of 12 mg ml^−1^ in 1 mM hydrochloric acid) was added to each well of a clear non-binding 96 well plate containing compound. His-tagged-rhCypD (50 nM in 35 mM HEPES pH 7.9) was added in equal volume to each well of the compound plate and incubated on ice for 30 minutes. Substrate (4.5 μl of N-Succinyl-Ala-Ala-Pro-Phe-p-nitroanalide; 5 mM in 450 mM lithium chloride) was dispensed in to a non-binding clear 96 well assay plate. The reaction was started through the addition of 90 μl of the chymotrypsin-rhCypD-compound solution to the assay plate and absorbance read at 405 nm using a Pherastar *FS* (BMG Labtech, Ortenberg, Germany). Data were collected at 3 second intervals over 60 seconds and area under the curve calculated. Data were normalised to CsA (2 μM; 100% inhibition) and DMSO (0% inhibition).

### NFAT-Luciferase immunosuppression

NFAT-RE-luc2 Jurkat cells were collected and centrifuged at 300 *g* and re-suspended in serum-free media containing supplements without antibiotics. Cell suspension (15,000 cells/20 μl) was added to each well of a solid black 384 well plate and incubated in the presence of compound for 45 minutes at 37 °C. A 3x solution containing ionomycin (final assay concentration 1 μM) and phorbol 12-myristate (PMA; final assay concentration 10 nM) was added to each well and cells incubated for 20 hours at 37 °C. An equal volume of Bright Glo reagent (Promega, Madison, WI) was added to all wells and plate allowed to stabilise for 10 minutes at room temperature. Luminescence was measured using the Pherastar *FS* (BMG Labtech, Ortenberg, Germany) and data normalised to CsA (5 μM; 100% inhibition) and DMSO (0% inhibition).

### Alamar Blue assay

NFAT-RE-luc2 Jurkat cells were collected, centrifuged at 300 *g* for 5 minutes and re-suspended in serum-free RPMI-1640 media, plus supplements without antibiotic. Cell suspension (15,000 cells/20 μl) was added to each well of a solid black 384 well plate and incubated in the presence of compound for 20 hours at 37 °C. Alamar Blue was used according to manufacturer’s instructions and was added to each well at 2x concentration in media and reaction allowed to proceed for 4 hours at 37 °C. Fluorescence (ex. 540 nm/em. 590 nm) was recorded using Pherastar *FS* (BMG Labtech, Ortenberg, Germany).

### CsA-CypD competition assay

A CypD homogeneous time resolved fluorescence (HTRF) binding assay was developed to assess the binding affinities of compounds of interest (Cisbio, Codolet, France). Functionalised CsA-NH_2_ was tagged with the d2-acceptor molecule. Binding of CsA-d2 to his-tagged CypD (Anti-His-Lumi4^®^-Tb cryptate donor) was measured through assessment of TR-FRET intensity between acceptor (CsA-d2) and donor molecules (Anti-His- Lumi4^®^-Tb). The following volumes of working stocks were added to a solid black 384 well plate containing 100 nl compound/DMSO: 10 μl/well cyclophilin D, 5 μl/well CsA-d2, 5 μl/well Anti-His- Lumi4^®^-Tb cryptate. Reagents were incubated for approximately 3 hours before fluorescence intensity (ex. 620 nm/em. 665 nm) was measured using a PheraStar *FS* (BMG Labtech, Ortenberg, Germany). Data were normalised to CsA (2 μM; 100% binding) and DMSO (0% binding).

### Experimental design, data analysis and statistical procedures

Data is presented as mean ± standard deviation (s.d.). Normalisation of the data allowed for control of inter-assay variability. Assay quality was determined after calculation of Z′^43^, using the equation: 
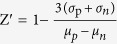
, taking into account the standard deviation (σ) and means (μ) of the positive (p) and negative (n) controls. Z′ > 0.35 was considered a pass. Curve fitting was performed using GraphPad Prism version 6.05 for Windows (La Jolla, California, USA). Statistical tests are indicated in figure legends. Statistical significance was assessed as being *P* < 0.05.

## Additional Information

**How to cite this article**: Briston, T. *et al*. Identification of ER-000444793, a Cyclophilin D-independent inhibitor of mitochondrial permeability transition, using a high-throughput screen in cryopreserved mitochondria. *Sci. Rep.* **6**, 37798; doi: 10.1038/srep37798 (2016).

**Publisher's note:** Springer Nature remains neutral with regard to jurisdictional claims in published maps and institutional affiliations.

## Figures and Tables

**Table 1:** Screening window for Ca^2+^ retention assay using cryopreserved mitochondria.

Months post preparation	Z′
Month 1	0.65
Month 2	0.73
Month 3	0.70
Month 4	0.54
Month 5	0.37
Month 6	0.54
Month 7	0.43

Z′ prime values were calculated as described in the methods. Over a 7 month period post-mitochondrial preparation, average Z′ prime values per month ranged from 0.37 to 0.73 in the Ca^2+^ retention assay.

**Table 2:** Summary data derived from the three screening assays.

	Swelling		TMRM		Calcium Retention	
	IC_50_ (M)	CI (95%)	IC_50_ (M)	CI (95%)	IC_50_ (M)	CI (95%)
ER-000444793	1.3E-05	4.0E-06 to 4.2E-05	2.8E-06	2.1E-06 to 3.6E-06	1.2E-06	9.5E-07 to 1.4E-06
CsA	2.0E-07	1.8E-07 to 2.3E-07	7.2E-08	6.6E-08 to 7.9E-08	3.4E-08	2.8E-08 to 4.2E-08

Data presented as mean IC_50_, calculated from three independent experiments and 95% confidence interval (CI).

**Table 3:** Calculated IC_50_ values from both human and rat liver mitochondria in the presence of ER-000444793 and CsA.

	IC_50_ Calcium Retention			
	ER-000444793		CsA	
	**IC**_**50**_ **(M)**	**CI (95%)**	**IC**_**50**_ **(M)**	**CI (95%)**
**Rat**	1.20E-06	9.5E-07 to 1.4E-06	3.40E-08	2.8E-08 to 4.2E-08
**Human**	2.00E-06	1.5E-06 to 2.7E-06	1.20E-07	9.3E-08 to 1.4E-07

Data presented as mean IC_50,_ calculated from three independent experiments and 95% confidence interval (CI).
